# Total Polyphenol Content and Antioxidant Capacity of Rosehips of Some *Rosa* Species

**DOI:** 10.3390/medicines5030084

**Published:** 2018-08-04

**Authors:** Noémi Koczka, Éva Stefanovits-Bányai, Attila Ombódi

**Affiliations:** 1 Institute of Horticulture, Szent István University, Páter K. street 1, 2100 Gödöllő, Hungary; ombodi.attila@mkk.szie.hu; 2 Department of Applied Chemistry, Szent István University, Villányi street 29-43, 1118 Budapest, Hungary; banyai.eva@etk.szie.hu

**Keywords:** phenolics, antioxidant activity, FRAP, *Rosa* spp., rosehip

## Abstract

**Background:** Rosehips, the fruits of *Rosa* species, are well known for their various health benefits like strengthening the immune system and treating digestive disorders. Antioxidant, anti-inflammatory, and cell regenerative effects are also among their health enhancing impacts. Rosehips are rich in compounds having antioxidant properties, like vitamin C, carotenoids, and phenolics. **Methods:** Total polyphenol content (Folin-Ciocalteu’s method), and in vitro total antioxidant capacity (ferric-reducing ability of plasma, FRAP) in rosehips of four *Rosa* species (*R. canina*, *R. gallica*, *R. rugosa*, *R. spinosissima*) were determined and compared. Ripe fruits were harvested at two locations. Water and ethanolic extracts of dried fruit flesh were analyzed. **Results:** *R. spinosissima* had the highest total phenolic content and antioxidant capacity, significantly higher than the other investigated *Rosa* species. Both parameters were reported in decreasing order for *R. spinosissima* > *R. canina* > *R. rugosa* > *R. gallica.* Ethanolic extracts of rosehips showed higher phenolic content and antioxidant activity than water extracts. Antioxidant properties were influenced by the growing site of *Rosa* species. **Conclusions:** This study indicates that *R. spinosissima* exhibited the greatest phenolic and antioxidant content, and therefore can be used as a reliable source of natural antioxidants, and serve as a suitable species for further plant breeding activities. Furthermore, investigations of various *Rosa* species for their antioxidant properties may draw more attention to their potential as functional foods.

## 1. Introduction

The genus *Rosa* contains more than 100 species which are widely distributed across Europe, temperate Asia, and North America [1,2]. Roses have been cultivated since ancient times, but some of them can still be found growing in the wild. They are climbing or bushy woody perennials with thorny stems and attractive, sweetly scented flowers of various colors [3]. Fleshy red fruits varying in shape and size are known as rosehip. Rose leaves, flowers, and fruits have been used for thousands of years for their medicinal benefits. The leaves have antioxidant and anti-inflammatory properties. Rose flowers have antibacterial, astringent, tonic, and antioxidant effects used for mild inflammation of the skin or lining of the mouth and throat [4]. Fruits can be consumed fresh, but they are mostly prepared as herbal tea, jam, jelly, syrup or wine. Rosehip has traditionally been used against a wide range of ailments due to its biological activities like immunosuppressive, antioxidant, anti-inflammatory, anti-arthritic, analgesic, anti-diabetic, cardioprotective, antimicrobial, gastroprotective, and skin ameliorative effects [5,6,7,8]. 

Rosehip contains the highest amount of vitamin C among fruits and vegetables, and also contains vitamin A, B_1_, B_2_, B_6_, D, E, and K [9,10,11,12]. Besides ascorbic acid, citric acid, and malic acid are the characteristic organic acids of the fruit [13,14]. Rosehip is also rich in carotenoids; lycopene, *ß*-cryptoxanthin, *ß*-carotene, rubixanthin, gazaniaxanthin, and zeaxanthin are identified as its major components [5,10,15]. Active ingredients of rosehip are furthermore pectin and sugars, mainly glucose and fructose [5,11,14]. Rosehip’s essential oil contains alcohols, aldehydes, monoterpenes, sesquiterpenes, and esters. The most abundant components are vitispiran, *α*-E-acaridial, hexadecanoic acid, docosane (C22), *ß*-ionone, 6-methyl-5-hepten-2-one, 2-heptanone, heptanal, and myristic acid [14,16]. Rosehip seeds have a high content of polyunsaturated fatty acids, the dominant compounds are linoleic acid (45–55%), followed by *α*-linolenic acid (18–32%) and oleic acid (13–20%) [17,18,19]. Rosehip contains different mineral nutrients, mainly phosphorus, potassium, calcium, magnesium, manganese, and zinc. The mineral composition of rosehips is highly dependent on species and environmental conditions [12].

Phenolic compounds including tannins, flavonoids, phenolic acids, and anthocyanins proved to be a very important group of biologically active ingredients present in rosehip [20]. Phenolics are well known for their antioxidant properties and there are a few studies analyzing the content and composition of polyphenols in different *Rosa* species, especially in *R. canina*. However, literature cites variable quantitative and qualitative descriptions of the phenolic profile of roses. Tumbas et al. [9] and Hosni et al. [21] identified quercetin and ellagic acid as the major phenolics of *R. canina*, while Türkben et al. [22] and Olsson et al. [23] reported quercetin and catechin to be the most important phenolic components in the species with an absence of ellagic acid or kaempferol. Demir et al. [14] and Elmastas et al. [24] identified phenolic acids in rosehip including gallic acid, 4-hydroxy benzoic acid, caftaric acid, 2,5-dihidroxy benzoic acid, chlorogenic acid, t-caffeic acid, p-coumaric acid, and ferrulic acid. Nadpal et al. [25] found protocatechuic acid in addition to the previously mentioned ones. The main flavonoids are methyl gallat, catechin [14,24], epicatechin [14,24,25], rutin, eriocitrin, quercetin, apigenin-7-*O*-glucoside, kaempferol, [14,24], quercitrin and, quinic acid [25].

Ercisli [12] reported a comprehensive study on the chemical composition of the species *R. canina*, *R. dumalis* subsp. *boissieri*, *R. dumalis* subsp. *antalyensis*, *R. villosa*, *R. pulverulenta*, and *R. pisiformis*, detecting the greatest total phenolic content in *R. canina*. Adamczak et al. [13] compared the flavonoid content of 11 *Rosa* species (*R. agrestis*, *R. canina*, *R. dumalis*, *R. glauca*, *R. inodora*, *R. jundzillii*, *R. rubiginosa*, *R. sherardii*, *R. tomentosa*, *R. villosa*, and *R. zalana*), finding a low average value of flavonoids for *R. canina*, the most common species, while flavonoids were the highest in *R. rubiginosa*. Demir et al. [14] investigated phenolic compounds of *R. canina*, *R. dumalis*, *R. gallica*, *R. dumalis* subsp. *boissieri*, and *R. hirtissima*, concluding that total phenolic contents of rosehips were significantly influenced by the species, whereas total flavonoid content was measured to be similar in all the examined species. Najda and Buczkowska [26] studied the chemical composition of *Rosa* species *R. californica*, *R. × damascena, R. rugosa*, *R. spinosissima*, and *R. villosa*. They found polyphenol content to be highly diverse in these species, with the highest total amount of phenolics measured in *R. rugosa* and *R. villosa*. Jimenez et al. [27] detected significant differences in total phenolic content among rosehips of *R. canina*, *R. corymbifera*, *R. glauca*, and *R. pouzinii* originating from different geographical zones. Nadpal et al. [25] found the total phenolics of *R. canina* to be significantly higher than that of *R. arvensis*. 

Hence, although wild grown and cultivated *Rosa* species and cultivars differ in their chemical composition and health promoting benefits, they can be considered a potential raw material for functional foods [26]. The most abundant and studied species is *R. canina*, called dog rose. It is native to Europe and Asia and it is naturalized in North America. Fruits are smooth, bright red-orange and 15–30 mm long. They persist on the plant for several months and become black [28]. Being the most collected *Rosa* taxon, its hips or hip extracts are added to vitamin C tablets, food supplements, herbal remedies, and herbal teas. Specimens of the plant are used as rootstocks for grafting. The wild plant itself is widely used to stabilize soil in land reclamation and specialized landscaping schemes [3] (p. 346) [29]. 

*R. gallica*, French rose, or apothecary rose is indigenous to Southern and Central Europe and the Caucasus. An outstanding number of cultivars were bred from this species by means of crossing. The species is cultivated for the petals which are used to extract essential oil or to prepare herbal medicines [30]. Hips are globose to ovoid, 10–13 mm in diameter, bristly with a color of brick-red to brownish. Fruits are mainly used in Ayurvedic medicine [3] (p. 347). 

*R. rugosa*, or Japanese rose is native to the Orient but the species has a wide range of adaptability. Due to the ability to hybridize with many other roses, and its high resistance to diseases like rose rust and rose black spot, it is a very important species for breeding processes. It is also remarkably tolerant to cold and salinity. Japanese rose is also a very popular plant material in landscaping as it is rather tolerant to environmental effects. Its rosehips are large, 20–30 mm in diameter and often slightly flat [31]. 

*R. spinosissima*, burnet rose, or Scots rose is endemic to Europe, and Western and Central Asia. Its hips are small (5–15 mm), globose or depressed globose with a black or dark purple color. Its cultivars are highly cold hardy and resistant to drought and diseases [32].

Several *in vitro* assays exist to measure the antioxidant capacity of food and biological samples. The sensitivity of these methods depends on some factors, such as pH, the presence of lipophilic and/or hydrophilic compounds. The ferric reducing ability of plasma (FRAP) assay is a simple and inexpensive method, however, effectively used for the detection of quantitative differences among samples. [33]. As reported earlier by several authors, the plant genotype, growing site, and extraction technique as well as differences in fruit ripeness, influence the total phenolic content and antioxidant activity of fruits [34,35,36,37].

The aim of this study was to determine and compare the total polyphenol content and total antioxidant capacity of rosehips of four *Rosa* species (*R. canina*, *R. gallica*, *R. rugosa*, and *R. spinosissima*). Total phenolics and FRAP values were evaluated both in water and ethanolic extracts of dried rosehips originating from two locations for each species. 

## 2. Materials and Methods 

### 2.1. Plant Material

Four *Rosa* species (*R. canina*, *R. gallica*, *R. rugosa*, and *R. spinosissima*) were selected for the study. Plants were located in Gödöllő (location 1, Northern Hungary) and in Szeged (location 2, Southern Hungary). Triplicated samples (100 g) of rosehips uniform in shape and color were collected randomly from different parts of the bushes at the ripe stage (with hard pericarp). Seeds were removed and analyses were carried out on fruit flesh. Samples were air-dried at 30 °C, then pulverized.

### 2.2. Extraction

Extraction was carried out according to Pharmacopoea Hungarica (Ph.Hg.) [38]. One hundred milliliters of distilled water was added to 1.00 g of dried material to prepare water extracts. Infusions were steeped for 24 h. The same sample weight and solvent volume were used for making ethanolic extracts with aqueous ethanol (water/ethanol 80/20 *v/v*, 20 °C), followed by a 72 h storage at room temperature. Extractions were replicated three times. Extracts were filtered and centrifuged at 1300 rpm for 10 min, then the supernatants were analyzed.

### 2.3. Determination of Total Polyphenols

For the determination of total phenolic content (TPC) by Folin-Ciocalteu reagent, the method described by Singleton and Rossi [39] was used. Briefly, 0.05 mL of diluted extract and 0.45 mL of distilled water were added to 2.5 mL of 1:10 diluted Folin-Ciocalteu’s phenol reagent, followed by the addition of 2 mL of 7.5% (*w*/*v*) sodium carbonate. After storing the solutions for 5 min at 50 °C, their absorbance was determined by spectrophotometer at 760 nm. TPC was estimated from a standard curve of gallic acid. All measurements were repeated three times and results were expressed as mg gallic acid equivalent (GAE) per 100 g dry weight (DW).

### 2.4. Determination of Antioxidant Capacity 

FRAP assay was carried out according to the method of Benzie and Strain [40] to characterize the antioxidant capacity of rosehip samples. This procedure is based on the reduction of ferric-tripyridyl-triazine (Fe^3+^-TPTZ) complex to the ferrous (Fe^2+^) form at low pH. Samples containing 100 μL of rosehip extract and 3 mL of FRAP solution were incubated at 37 °C for 4 min, then their absorbance was measured at 593 nm. Change in the absorbance compared to that of the standard solution of L-ascorbic acid (AA) was converted into a FRAP value, and the result was expressed as mmol AA per g DW.

### 2.5. Statistical Analysis

Data were analyzed using Microsoft Excel software. The effect of extraction method was investigated by paired *t*-tests. Effects of species and collection site were investigated by two-way analysis of variance performed separately for water extraction and for ethanol extraction data. Fisher’s least significant difference test was applied as a post-hoc test.

## 3. Results

### 3.1. Total Polyphenol Content

**Figure 1:**
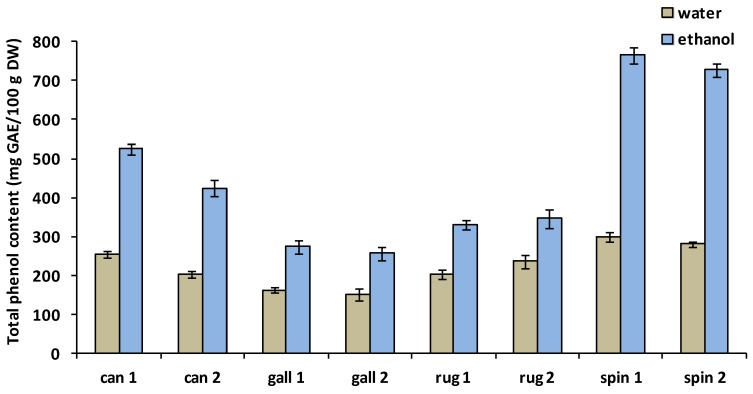
Total polyphenol content of water and ethanolic extracts of *R. canina* (can), *R. gallica* (gall), *R. rugosa* (rug) and *R. spinosissima* (spin); means ± SD; 1 = location 1 (Gödöllő), 2 = location 2 (Szeged). GAE: gallic acid equivalent; DW: dry weight.

The total phenolic content (TPC) was determined both from water and ethanolic extracts from hips of selected *Rosa* species. Results are summarized in Figure 1. Water soluble TPC values for analyzed rosehips ranged from 150.8 mg to 299.2 mg GAE/100 g DW. *R. spinosissima* was characterized by the highest phenolic content. Significantly lower values were found both for *R. canina* and *R. rugosa* which showed similar values. Significantly, the lowest TPC level was measured for *R. gallica*. In water extract of *R. canina*, TPC level proved to be significantly higher in the samples from Gödöllő (location 1) than in those from Szeged (location 2). However, there were no remarkable differences between the two sampling sites in the case of the other three species. 

The ethanolic extraction method resulted in significantly higher TPC values for all investigated rose species compared to aqueous extraction. TPC in ethanolic extracts varied from 255.9 mg to 766.0 mg GAE/100 g DW (Figure 1). Differences among the four investigated species were significant. Similarly to aqueous extraction, the highest TPC value was found in *R. spinosissima*, three fold higher than values of *R. gallica*, characterized by the lowest phenolic content. TPC levels in *R. canina* were significantly higher than those in *R. rugosa* and *R. gallica*. Significant differences between collecting sites were obtained only for *R. canina*: location 1 showed higher TPC values in ethanolic extracts than those of location 2.

### 3.2. Antioxidant Capacity

**Figure 2:**
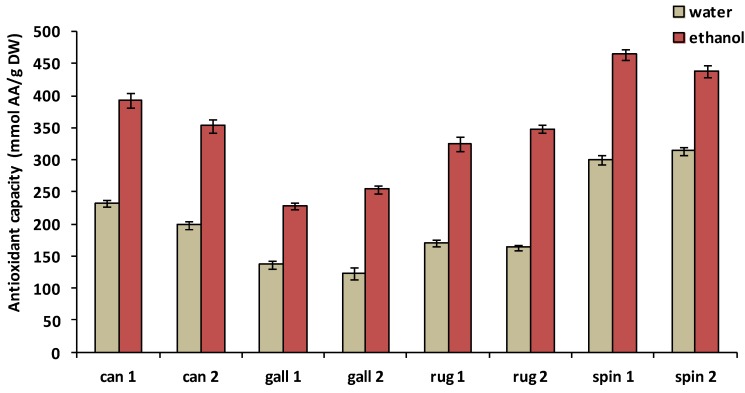
Antioxidant capacity (FRAP) of water and ethanolic extracts of *R. canina* (can), *R. gallica* (gall), *R. rugosa* (rug) and *R. spinosissima* (spin); means ± SD; 1 = location 1 (Gödöllő), 2 = location 2 (Szeged). AA: ascorbic acid.

Antioxidant capacity was measured using FRAP assay, values for water and ethanolic extracts are represented in Figure 2. The FRAP values, expressed as ascorbic acid equivalents per g DW, varied from 123.8 mmol to 314.4 mmol in water extracts. The highest FRAP values were noted for *R. spinosissima*, followed by *R. canina*, then *R. rugosa*, while the lowest values were detected in *R. gallica*. Differences among the species were found to be statistically significant. FRAP in water extracts was only influenced by the growing site in the case of *R. canina*.

Ethanolic extracts showed significantly higher antioxidant capacities than water extracts in all four *Rosa* species (Figure 2). FRAP values of ethanolic extracts of rosehips ranged from 228.2 mmol to 464.8 mmol AA/g DW. The highest antioxidant capacity was detected for *R. spinosissima*, two-fold higher than data obtained for *R. gallica*, representing the lowest values. FRAP values measured in ethanolic extracts of *R. canina* and *R. rugosa* were significantly lower than those of *R. spinosissima*, whereas no significant difference between *R. canina* and *R. rugosa* was detected. Ethanolic extracts of *R. spinosissima* and *R. canina* had significantly higher antioxidant properties in samples originating from Gödöllő (location 1) than those from Szeged (location 2). However, in the case of *R. gallica* and *R. rugosa*, location 2 was characterized by higher FRAP values than location 1.

## 4. Discussion

In the present study, total phenol content of water and ethanolic extracts from rosehips of different species were evaluated. Comparing the two extraction methods, ethanolic extracts showed significantly higher TPC values than water extracts in all cases. Detected ranges of TPC are in agreement with some earlier studies, although with slight quantitative differences. For *R. canina*, Roman et al. [41] found a TPC range from 326 mg to 575 mg GAE/100 g DW, Yoo et al. [42] measured 818 mg GAE/100 g DW in water extracts, while Fattahi et al. [43] measured 180–225 mg GAE/100 g DW, Yilmaz and Ercisli [44] 102 mg GAE/100 g DW, and Barros et al. [45] 149.35 mg GAE/g extract in methanolic extracts. On the other hand, values over ten-fold higher than our findings were reported by Ercisli [12] (9600 mg GAE/100 g DW) and Demir et al. [14] (3108 mg GAE/100 g DW) in water extracts, and by Nadpal et al. [25] (6100 mg GAE/100 g DW in water, 5030 mg GAE/100 g DW in methanolic extract) for the same species. Najda and Buczkowska [26] obtained very low TPC levels: 215.14 mg GAE/100 g fresh weight for *R. rugosa*, and 121.38 mg GAE/100 g fresh weight for *R. spinosissima*. Much higher TPC was noted by Demir et al. [14] for *R. gallica* (3151 mg GAE/100 g DW) than measured in the present study. 

Both extraction methods revealed significant differences in total phenolic content among the investigated species. The concentration of TPC was obtained in a decreasing order of *R. spinosissima* > *R. canina* > *R. rugosa* > *R. gallica.* In this study, *R. spinosissima* was responsible for the highest TPC value compared to the other three species. Fattahi et al. [43] evaluated similar values for *R. spinosissima* and *R. canina*, while Najda and Buczkowska [26] found significantly lower TPC level for *R. spinosissima* than for *R. rugosa*. Demir et al. [14] reported no differences between TPC content of *R. canina* and *R. gallica*. 

Rosehips of four *Rosa* species were collected at the same time at two locations. Growing site had no determining effect on TPC and in the case of phenolic content it only significantly affected the results of *R. canina*, but not the other three investigated species. 

Total antioxidant capacity was evaluated by using FRAP assay, both in water and ethanolic extracts. The obtained ranges of FRAP values proved to be similar to those found by Gao et al. [46], Demir et al. [14], Taneva et al. [47] and Nadpal et al. [25], but much higher than those reported by Koca et al. [48]. Other antioxidant capacity determination methods than FRAP are also frequently used in rosehip experiments. Barros et al. [45] and Tumbas et al. [9] used DPPH (1,1-diphenyl-2-picrylhydrazyl) radical scavenging activity, while Montazeri et al. [49] applied DPPH and ABTS (2,2′-azinobis-3-ethylbenzothiazoline-6-sulfonic acid) to characterize antioxidant properties of *R. canina*. Fattahi et al. [43] determined the antioxidant capacity of *R. canina* and *R. spinosissima* by DPPH assay and hydrogen peroxide (H_2_O_2_) radical scavenging assay. Franco et al. [50] and Olech et al. [51] investigated rosehip antioxidants using DPPH for *R. rubiginosa* and for *R. rugosa*, respectively, while Najda and Buczkowska [26] gained extracts with DPPH for *R. californica*, *R. damascena*, *R. rugosa* and *R. villosa*. 

It is worth underlining the effect of different solvents on total phenol content and antioxidant activity. In this study, ethanolic extracts had markedly higher antioxidant capacities than those of water extracts. Therefore ethanol is a more effective solvent for extraction of antioxidant compounds of *Rosa* species. This result is in agreement with the findings of Taneva et al. [47] and Franco et al. [50]. Ilbay et al. [52] found methanol extraction three-fold more effective than water extraction. However, Olech et al. [51] reported that *R. rugosa* ethanol extract had an antioxidant activity similar to that of water extract. Nadpal et al. [25] found that methanol is a more effective solvent for the extraction of phenolic compounds than water in the case of *R. canina* but obtained just the opposite for *R. arvensis*. Higher phenolic levels and antioxidant capacity were found in methanolic and/or ethanolic extracts of other plant species compared to water extracts by some authors [53,54,55,56]. Therefore in recent scientific studies, alcoholic extraction is more frequently used than water extraction to determine antioxidant properties of different plant materials [57,58,59,60]. 

High variability was found in FRAP values among the four species. *R. spinosissima* was responsible for much higher antioxidant activity than the other species. FRAP values were detected, similarly to TPC, in a decreasing order of *R. spinosissima* > *R. canina* > *R. rugosa* > *R. gallica.* Our data showed that *R. spinosissima* exhibited the greatest total phenolic content and total antioxidant capacity among the four studied *Rosa* species. This result indicates that *R. spinosissima* can be used as a reliable source of natural antioxidants. Based on the findings, this species is highly recommended as a breeding material for medicinal purposes. In the past, several *R. spinosissima* varieties were cultivated, mainly in Europe. However, today only few remained, so this species became an underexploited genetic resource [32]. Investigation of various *Rosa* species for their antioxidant properties may draw more attention to their potential as a functional food or food additive.

Antioxidant capacity was affected by the growing location of the rosehip: FRAP values obtained in ethanolic extracts were significantly different in samples from the two sites in the case of all the investigated species. FRAP values of water extracts differed markedly only for *R. canina*. This result suggests that the antioxidant activity of *R. canina* is strongly dependent on environmental factors. As shown in Figure 2, antioxidant capacity of ethanolic extracts of *R. canina* and *R. spinosissima* was higher in samples from location 1, however, the opposite was found for *R. gallica* and *R. rugosa*. These differences indicate that the growing site influences the antioxidant properties depending on the species. In our case, location 2 is characterized by slightly higher temperatures and more sunshine hours in the vegetation period than location 1. These climatic conditions seem to favor the forming of antioxidant components in the case of *R. gallica* and *R. rugosa*. 

As the results of this study demonstrate, antioxidant activity of different *Rosa* species is recommended to be analyzed more comprehensively. Furthermore, differences in the antioxidant properties among samples of the same species from different locations underline the importance of further investigations under different environmental conditions. The results also revealed that different solvents and extraction methods should also be examined as they play an important role in biological activity. The concentration of extracted bioactive ingredients greatly influences the medicinal effects of the rosehip.
